# Computational Neuropsychology and Bayesian Inference

**DOI:** 10.3389/fnhum.2018.00061

**Published:** 2018-02-23

**Authors:** Thomas Parr, Geraint Rees, Karl J. Friston

**Affiliations:** ^1^Wellcome Trust Centre for Neuroimaging, Institute of Neurology, University College London, London, United Kingdom; ^2^Institute of Cognitive Neuroscience, University College London, London, United Kingdom

**Keywords:** neuropsychology, active inference, predictive coding, computational phenotyping, precision

## Abstract

Computational theories of brain function have become very influential in neuroscience. They have facilitated the growth of formal approaches to disease, particularly in psychiatric research. In this paper, we provide a narrative review of the body of computational research addressing neuropsychological syndromes, and focus on those that employ Bayesian frameworks. Bayesian approaches to understanding brain function formulate perception and action as inferential processes. These inferences combine ‘prior’ beliefs with a generative (predictive) model to explain the causes of sensations. Under this view, neuropsychological deficits can be thought of as false inferences that arise due to aberrant prior beliefs (that are poor fits to the real world). This draws upon the notion of a Bayes optimal pathology – optimal inference with suboptimal priors – and provides a means for computational phenotyping. In principle, any given neuropsychological disorder could be characterized by the set of prior beliefs that would make a patient’s behavior appear Bayes optimal. We start with an overview of some key theoretical constructs and use these to motivate a form of computational neuropsychology that relates anatomical structures in the brain to the computations they perform. Throughout, we draw upon computational accounts of neuropsychological syndromes. These are selected to emphasize the key features of a Bayesian approach, and the possible types of pathological prior that may be present. They range from visual neglect through hallucinations to autism. Through these illustrative examples, we review the use of Bayesian approaches to understand the link between biology and computation that is at the heart of neuropsychology.

## Introduction

The process of relating brain dysfunction to cognitive and behavioral deficits is complex. Traditional lesion-deficit mapping has been vital in the development of modern neuropsychology but is confounded by several problems (Bates et al., 2003). The first is that there are statistical dependencies between lesions in different regions (Mah et al., 2014). These arise from, for example, the vascular anatomy of the brain. Such dependencies mean that regions commonly involved in stroke may be spuriously associated with a behavioral deficit (Husain and Nachev, 2007). The problem is further complicated by the distributed nature of brain networks (Valdez et al., 2015). Damage to one part of the brain may give rise to abnormal cognition indirectly – through its influence over a distant region (Price et al., 2001; Carrera and Tononi, 2014). An understanding of the contribution of a brain region to the network it participates in is crucial in forming an account of functional diaschisis of this form (Boes et al., 2015; Fornito et al., 2015). Solutions that have been proposed to the above problems include the use of multivariate methods (Karnath and Smith, 2014; Nachev, 2015) to account for dependencies, and the use of models of effective connectivity to assess network-level changes (Rocca et al., 2007; Grefkes et al., 2008; Abutalebi et al., 2009; Mintzopoulos et al., 2009) in response to lesions.

In this paper, we consider a complementary approach that has started to gain traction in psychiatric research (Adams et al., 2013b, 2015; Corlett and Fletcher, 2014; Huys et al., 2016; Schwartenbeck and Friston, 2016; Friston et al., 2017c). This is the use of models that relate the computations performed by the brain to measurable behaviors (Krakauer and Shadmehr, 2007; Mirza et al., 2016; Testolin and Zorzi, 2016; Iglesias et al., 2017). Such models can be associated with process theories (Friston et al., 2017a) that map to neuroanatomy and physiology. This complements the approaches outlined above, as it allows focal neuroanatomical lesions to be interpreted in terms of their contribution to a network. Crucially, this approach ensures that the relationship between brain structure and function is addressed within a conceptually rigorous framework – this is essential for the construction of well-formed hypotheses for neuropsychological research (Nachev and Hacker, 2014). We focus here upon models that employ a conceptual framework based on Bayesian inference.

Bayesian inference is the process of forming beliefs about the causes of sensory data. It relies upon the combination of prior beliefs about these causes, and beliefs about how these causes give rise to sensations. Using these two probabilities it is possible to calculate the probability, given a sensation, of its cause. This is known as a ‘posterior’ probability. This means that prior beliefs are updated by a sensory experience to become posterior beliefs. These posteriors can then be used as the prior for the next sensory experience. In short, Bayesian theories of brain function propose the brain encodes beliefs about the causes of sensory data, and that these beliefs are updated in response to new sensory evidence.

Our motivation for pursuing a Bayesian framework is that it captures many different types of behavior, including apparently suboptimal behaviors. According to an important result known as *the complete class theorem* (Wald, 1947; Daunizeau et al., 2010), there is always a set of a prior belief that renders an observed behavior Bayes optimal. This is fundamental for computational neuropsychology as it means we can cast even pathological behaviors as the result of processes that implement Bayesian inference (Schwartenbeck et al., 2015). In other words, we can assume that the brain makes use of a probabilistic model of its environment to make inferences about the causes of sensory data (Knill and Pouget, 2004; Doya, 2007), and to act upon them (Friston et al., 2012b). Another consequence of the theorem is that computational models that are not (explicitly) motivated by Bayesian inference (Frank et al., 2004; O’Reilly, 2006) may be written down in terms of Bayesian decision processes. Working within this framework facilitates communication between these models, and ensures they could all be used to phenotype patients using a common currency (i.e., their prior beliefs). It follows that the key challenges for computational neuropsychology can be phrased in terms of two questions: ‘what are the prior beliefs that would have to be held to make this behavior optimal?’ and ‘what are the biological substrates of these priors?’

The notion of optimal pathology may seem counter-intuitive, but we can draw upon another theorem, *the good regulator theorem* (Conant and Ashby, 1970), to highlight the difference between healthy and pathological behavior. This states that a brain (or any other system) is only able to effectively regulate its environment if it is a good model of that environment. A brain that embodies a model with priors that diverge substantially from the world (i.e., body, ecological niche, culture, etc.) it is trying to regulate will fail at this task (Schwartenbeck et al., 2015). If pathological priors relate to the properties of the musculoskeletal system, we might expect motor disorders such as tremors or paralysis (Friston et al., 2010; Adams et al., 2013a). If abnormal priors relate to perceptual systems, the results may include sensory hallucinations (Fletcher and Frith, 2009; Adams et al., 2013b) or anesthesia. In the following, we review some important concepts in Bayesian accounts of brain function. These include the notion of a generative model, the hierarchical structure of such models, the representation of uncertainty in the brain, and the active nature of sensory perception. In doing so we will develop a taxonomy of pathological priors. While this taxonomy concerns types of inferential deficit (and is not a comprehensive review of neuropsychological syndromes), we draw upon examples of syndromes to illustrate these pathologies. We relate these to failures of neuromodulation and to the notion of a ‘disconnection’ syndrome (Geschwind, 1965a; Catani and Ffytche, 2005).

## The Generative Model

### Bayesian Inference

**FIGURE 1:**
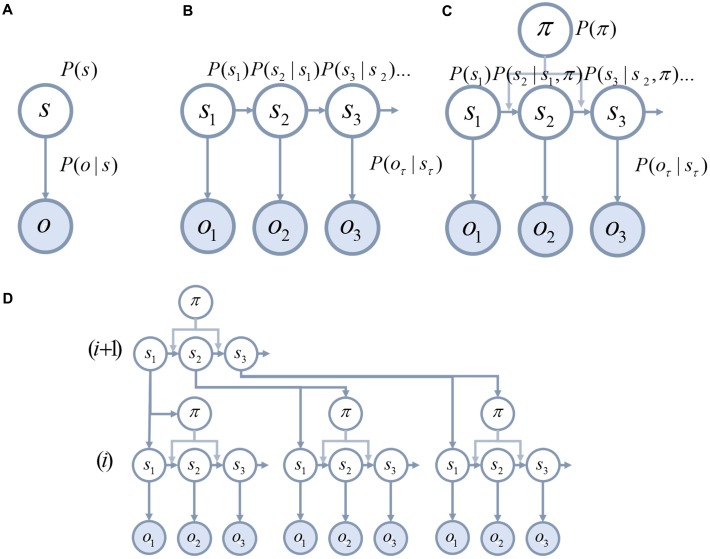
Generative models. These schematics graphically illustrate the structure of generative models. **(A)** The simplest model that permits Bayesian inference involves a hidden state, *s*, that is equipped with a prior *P*(*s*). This hidden state generates observable data, *o*, through a process defined by the likelihood *P*(*o*|*s*) (vertical arrow). **(B)** It is possible to equip such a model with dynamically changing hidden states. To do so, we must specify the probabilities of transitioning between states *P*(*s*_τ+1_|*s*_τ_) (horizontal arrows). **(C)** Transitions between states may be influenced by the course of action, π, that is pursued. **(D)** Hierarchical levels can be added to the generative model (Friston et al., 2017d). This means that the processes that generate the hidden states can themselves be accommodated in the inferences performed using the model.

Much work in theoretical neurobiology rests on the notion that the brain performs Bayesian inference (Knill and Pouget, 2004; Doya, 2007; Friston, 2010; O’Reilly et al., 2012). In other words, the brain makes inferences about the (hidden or latent) causes of sensory data. ‘Hidden’ variables are those that are not directly observable and must be inferred. For example, the position (hidden variable) of a lamp causes a pattern of photoreceptor activation (sensory data) in the retina. Bayesian inference can be used to infer the probable position of the lamp from the retinal data. To do this, two probability distributions must be defined (these are illustrated graphically in **Figure 1A**). These are the prior probability of the causes, and a likelihood distribution that determines how the causes give rise to sensory data. Together, these are referred to as a ‘generative model,’ as they describe the processes by which data is (believed to be) generated. Bayesian inference uses a generative model to compute the probable causes of sensory data (Beal, 2003; Doya, 2007; Ghahramani, 2015). Many of the inferences that must be made by the brain relate to causes that evolve through time. This means that the prior over the *trajectory* of causes through time can be decomposed into a prior for the initial state, and a series of transition probabilities that account for sequences or dynamics (**Figure 1B**). These dynamics can be subdivided into those that a subject has control over (**Figure 1C**), such as muscle length, and environmental causes that they cannot directly influence.

### Predictive Coding

Predictive coding is a prominent theory describing how the brain could perform Bayesian inference (Rao and Ballard, 1999; Friston and Kiebel, 2009; Bastos et al., 2012). This relies upon the idea that the brain uses its generative model to form perceptual hypotheses (Gregory, 1980) and make predictions about sensory data. The difference between this prediction and the incoming data is computed, and the ensuing prediction error is used to refine hypotheses about the cause of the data. Under this theory, the messages passed through neuronal signaling are either predictions, or prediction errors. There are other local message passing schemes that can implement Bayesian inference (Winn and Bishop, 2005; Yedidia et al., 2005; Dauwels, 2007; Friston et al., 2017b), particularly for categorical (as opposed to continuous) inferences. Although we use the language of predictive coding in the following, we note that our discussion generalizes to other Bayesian belief propagation schemes.

The notion that hypotheses are corrected by prediction errors makes sense of the kinds of neuropsychological pathologies that result from the loss of sensory signals. For example, patients with eye disease can experience complex visual hallucinations (Ffytche and Howard, 1999). This phenomenon, known as Charles Bonnet syndrome (Teunisse et al., 1996; Menon et al., 2003), can be interpreted as a failure to constrain perceptual hypotheses with sensations (Reichert et al., 2013). In other words, there are no prediction errors to correct predictions. A similar line of argument can be applied to phantom limbs (Frith et al., 2000; De Ridder et al., 2014). Following amputation, patients may continue to experience ‘phantom’ sensory percepts from their missing limb. The absence of corrective signals from amputated body parts means that any hypothesis held about the limb is unfalsifiable. In the next sections, we consider some of the important features of generative models, and their relationship to brain function.

## Hierarchical Models

### Cortical Architecture

An important feature of many generative models is hierarchy. Hierarchical models assume that the hidden causes that generate sensory data are themselves generated from hidden causes at a higher level in the hierarchy (**Figure 1D**). As the hierarchy is ascended, causes tend to become more abstract, and have dynamics that play out over a longer time course (Kiebel et al., 2008, 2009). An intuitive example is the kind of generative model required for reading (Friston et al., 2017d). While lower levels may represent letters, higher levels represent words, then sentences, then paragraphs.

There are several converging lines of evidence pointing to the importance of hierarchy as a feature of brain organization. One of these is the patterns of receptive fields in the cortex (Gallant et al., 1993). In primary sensory cortices, cells tend to respond to simple features such as oriented lines (Hubel and Wiesel, 1959). As we move further from sensory cortices, the complexity of the stimulus required to elicit a response increases. Higher areas become selective for contours (Desimone et al., 1985; von der Heydt and Peterhans, 1989), shapes, and eventually objects (Valdez et al., 2015). The sizes of receptive fields also increase (Gross et al., 1972; Smith et al., 2001).

**FIGURE 2:**
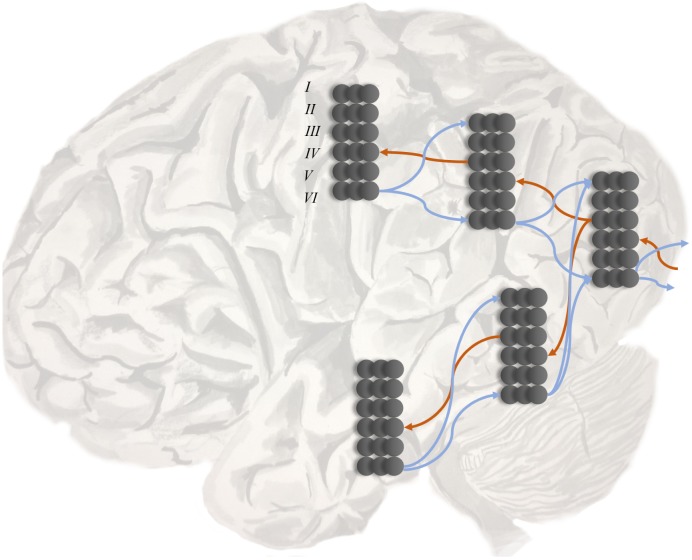
Hierarchy in the cortex. This schematic illustrates two key features of cortical organization. The first is hierarchy, as defined by laminar specific projections. Projections from primary sensory areas, such as area VI, to higher cortical areas typically arise from layer III of a cortical column, and target layer IV. These ascending connections are shown in red. In contrast, descending connections (in blue) originate in deep layers of the cortex and project to both superficial and deep laminae. The second feature illustrated here is the separation of visual processing into two, dorsal and ventral, streams. In terms of the functional anatomy implied by generative models in the brain, this segregation implies a factorization of beliefs about the location and identity of a visual object (i.e., knowing what an object is does not tell you where it is – and vice versa).

A second line of evidence is the change in temporal response properties. Higher areas appear to respond to stimuli that change over longer time courses than lower areas (Hasson et al., 2008, 2015; Kiebel et al., 2008; Murray et al., 2014). This is consistent with the structure of deep temporal generative models (Friston et al., 2017d) (a sentence takes longer to read than a word). A third line of evidence is the laminar specificity of inter-areal connections that corroborates the pattern implied by electrophysiological responses (Felleman and Van Essen, 1991; Shipp, 2007; Markov et al., 2013). As illustrated in **Figure 2**, cortical regions lower in the hierarchy project to layer IV of the cortex in higher areas. These ‘ascending’ connections arise from layer III of the lower hierarchical region. ‘Descending’ connections typically arise from deep layers of the cortex, and target both deep and superficial layers of the cortical area lower in the hierarchy.

### Ascending and Descending Messages

The parallel between the hierarchical structure of generative models and that of cortical organization has an interesting consequence. It suggests that connections between cortical regions at different hierarchical levels are the neurobiological substrate of the likelihoods that map hidden causes to the sensory data, or lower level causes, that they generate (Kiebel et al., 2008; Friston et al., 2017d). This is very important in understanding the computational nature of a ‘disconnection’ syndrome. It implies that the disruption of a white matter pathway corresponds to an abnormal prior belief about the form of the likelihood distribution. This immediately allows us to think of neurological disconnection syndromes – such as visual agnosia, pure alexia, apraxia, and conduction aphasia (Catani and Ffytche, 2005) – in probabilistic terms. We will address specific examples of these in the next section, and a summary is presented in **Table 1**. Under predictive coding, the signals carried by inter-areal connections have a clear interpretation (Shipp et al., 2013; Shipp, 2016). Descending connections carry the predictions derived from the generative model about the causes or data at the lower level. Ascending connections carry prediction error signals.

**Table 1:** Bayesian computational neuropsychology.

Syndrome	Abnormal prior	Neurobiology	Reference
Anosognosia	Low exteroceptive or interoceptive sensory precision Failure of *active* inference	Insula lesions Hemiplegia	Karnath et al., 2005; Fotopoulou et al., 2010; Fotopoulou, 2012
Apraxia	Disrupted likelihood (action to vision or command to action consequences)	Callosal disconnection Left frontoparietal disconnection	Geschwind, 1965b
Autism	High sensory precision	↑Cholinergic transmission?	Dayan and Yu, 2006; Lawson et al., 2014; Marshall et al., 2016; Lawson et al., 2017
	Secondary to high volatility	↑Noradrenergic transmission	
Complex visual hallucinations (Lewy body dementia, Charles Bonnet syndrome)	Low sensory precision Disrupted likelihood mapping	↓Cholinergic transmission Retino-geniculate disconnection	Collerton et al., 2005; Reichert et al., 2013; O’Callaghan et al., 2017
Conduction aphasia	Disrupted likelihood mapping (speech to proprioceptive consequences)	Arcuate fasciculus disconnection	Wernicke, 1969
Parkinson’s disease	Low prior precision over policies	↓Dopaminergic transmission	Friston et al., 2013
Visual agnosia	Disrupted likelihood (‘what’ to sensory data)	Ventral visual stream disconnection	Geschwind, 1965b
Visual neglect	Disrupted likelihood (fixation to ‘what’) mapping Biased outcome prior Biased policy prior	SLF II disconnection Pulvinar lesion Putamen lesion	Karnath et al., 2002; Thiebaut de Schotten et al., 2005; Bartolomeo et al., 2007

It has been argued that deficits in semantic knowledge can only be interpreted with reference to a hierarchically organized set of representations in the brain. This argument rests on observations that patients with agnosia, a failure to recognize objects, can present with semantic deficits at different levels of abstraction. For example, some neurological patients are able to distinguish between broad categories (fruits or vegetables) but are unable to identify particular objects within a category (Warrington, 1975). The preservation of the more abstract knowledge, with impairment of within-category semantics, is taken as evidence for distinct hierarchical levels that can be differentially impaired. This is endorsed by findings that some patients have a category-specific agnosia (for example, a failure to identify living but not inanimate stimuli) (Warrington and Shallice, 1984). A model that simulates these deficits relies upon a hierarchical structure that allows for specific categorical processing at higher levels to be lesioned while maintaining lower level processes (Humphreys and Forde, 2001). Notably, lesions to this model were performed by modulating the connections between hierarchical levels. This resonates well with the type of computational ‘disconnection’ that predictive coding implicates in some psychiatric disorders (Friston et al., 2016a). We now turn to the probabilistic interpretation of such disconnections.

## Sensory Streams and Disconnection Syndromes

### What and where?

**Figure 2** illustrates an additional feature common to cortical architectures and inference methods. This is the factorization of beliefs about hidden causes into multiple streams. Bayesian inference often employs this device, known as a ‘mean-field’ assumption, which ‘carves’ posterior beliefs into the product of statistically independent factors (Beal, 2003; Friston and Buzsáki, 2016). The factorization of visual hierarchies into ventral and dorsal ‘what’ and ‘where’ streams (Ungerleider and Mishkin, 1982; Ungerleider and Haxby, 1994) appears to be an example of this. A closely related factorization separates the dorsal and ventral attention networks (Corbetta and Shulman, 2002). This factorization has important consequences for the representation of objects in space. Location is represented bilaterally in the brain, with each side of space represented in the contralateral hemisphere. As it is not necessary to know the location of an object to know its identity, it is possible to represent this information independently, and therefore unilaterally (Parr and Friston, 2017a). It is notable that object recognition deficits tend to occur when patients experience damage to areas in the right hemisphere (Warrington and James, 1967, 1988; Warrington and Taylor, 1973). Lesions to contralateral (left hemispheric) homologs are more likely to give rise to difficulties in naming objects (Kirshner, 2003).

The bilateral representation of space has an important consequence when we frame neuronal processing as probabilistic inference. Following an inference that a stimulus is likely to be on one side of space, it must be the case that it is less likely to be on the contralateral side. If neuronal activities in each hemisphere represent these probabilities, this induces a form of interhemispheric competition (Vuilleumier et al., 1996; Rushmore et al., 2006; Dietz et al., 2014). An important role of commissural fiber pathways may be to enforce the normalization of probabilities across space [although some of these axons must represent likelihood mappings instead (Glickstein and Berlucchi, 2008)]. This neatly unifies theories that relate disorders of spatial processing to interhemispheric (Kinsbourne, 1970) or intrahemispheric disruptions (Bartolomeo et al., 2007; Bartolomeo, 2014). Any intrahemispheric lesion that induces a bias toward one side of space necessarily alters the interhemispheric balance of activity (Parr and Friston, 2017b).

### Disconnections and Likelihoods

The factorization of beliefs into distinct processing streams is not limited to the visual system. Notably, theories of the neurobiology of speech propose a similar division into dorsal and ventral streams (Hickok and Poeppel, 2007; Saur et al., 2008). The former is thought to support articulatory components of speech, while the latter is involved in language comprehension. This mean-field factorization accommodates the classical subdivision of aphasias into fluent (e.g., Wernicke’s aphasia) and non-fluent (e.g., Broca’s aphasia) categories. The anatomy of these networks has been interpreted in terms of predictive coding (Hickok, 2012a,b), and this interpretation allows us to illustrate the point that disconnection syndromes are generally due to disruption of the likelihood mapping between two regions. We draw upon examples of aphasic and apraxic syndromes to make this point.

**FIGURE 3:**
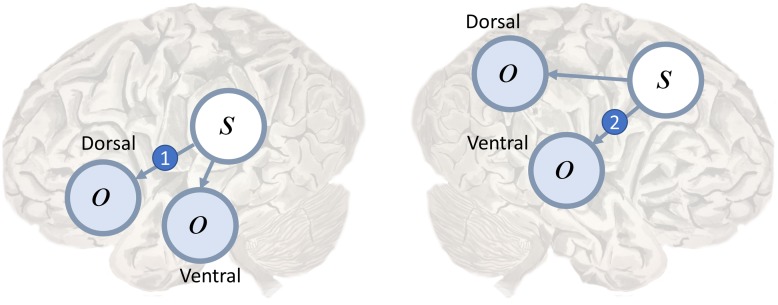
Dorsal and ventral streams. Here we depict a plausible mapping of simple generative models to the dual streams of the language **(Left)** and attention **(Right)** networks. We highlight the likelihood mappings that correspond to white matter tracts implicated in disconnection syndromes. The number 1 in the blue circle on the left highlights the mapping from the left temporoparietal region, which responds to spoken words (Howard et al., 1992), to the inferior frontal gyrus, involved in the dorsal articulatory stream (Hickok, 2012b). This region is well placed to deal with proprioceptive data from the laryngeal and pharyngeal muscles (Simonyan and Horwitz, 2011). The connection corresponds to the arcuate fasciculus and lesions give rise to conduction aphasia. The number 2 indicates the mapping from dorsal frontal regions that represent eye fixation locations to ventral regions associated with target detection and identity. This corresponds to the second branch of the superior longitudinal fasciculus. Lesions to this structure are implicated in visual neglect (Doricchi and Tomaiuolo, 2003; Thiebaut de Schotten et al., 2005).

Conduction aphasia is the prototypical disconnection syndrome (Wernicke, 1969), disconnecting Wernicke’s area from Broca’s area. The former is found near the temporoparietal junction, and is thought to contribute to language comprehension. The latter is in the inferior frontal lobe, and is a key part of the dorsal language stream. Disconnection of the two areas results in an inability to repeat spoken language. This connection between these two areas, the arcuate fasciculus (Catani and Mesulam, 2008), could represent the likelihood mapping from speech representations in Wernicke’s area to the articulatory proprioceptive data processed in Broca’s area as in **Figure 3** (left). While auditory data from the ventral pathway may inform inferences about language, the failure to translate these into proprioceptive predictions means that such predictions cannot be fulfilled by the brainstem motor system (Adams et al., 2013a).

The idea that a common generative model could generate both auditory and proprioceptive predictions, associated with speech, harmonizes well with theories of about the ‘mirror-neuron’ system (Di Pellegrino et al., 1992; Rizzolatti et al., 2001). These neurons respond both to the performance of an action by an individual, and when that individual observes the same action being performed by another. Similarly, Wernicke’s area appears to be necessary for both language comprehension and generation (Dronkers and Baldo, 2009) (but see Binder, 2015). Anatomically, there is consistency between the mirror neuron system and the connectivity between the frontal and temporal regions involved in speech. The former is often considered to include Broca’s area and the superior temporal sulcus – adjacent to Wernicke’s area (Frith and Frith, 1999; Keysers and Perrett, 2004).

A common generative model for action observation and generation (Kilner et al., 2007) generalizes to include the notion of ‘conduction apraxia’ (Ochipa et al., 1994). As with conduction aphasia, this disorder involves a failure to repeat what another is doing. Instead of repeating spoken language, conduction apraxia represents a deficit in mimicking motor behaviors. This implies a disconnection between visual and motor regions (Goldenberg, 2003; Catani and Ffytche, 2005). This must spare the route from language areas to motor areas. Other forms of apraxia have been considered to be disconnection syndromes in which language areas are disconnected from motor regions, preventing patients from obeying a verbal motor command (Geschwind, 1965b). Under this theory, deficits in imitation that accompany this are due to disruption of axons that connect visual and motor areas. These also travel in tracts from posterior to frontal cortices.

Other disconnection syndromes include (Geschwind, 1965a; Catani and Ffytche, 2005) visual agnosia, caused by disruption of connections in the ventral visual stream, and visual neglect (Doricchi and Tomaiuolo, 2003; Bartolomeo et al., 2007; He et al., 2007; Ciaraffa et al., 2013). Neglect can be a consequence of frontoparietal disconnections (**Figure 3**, right), leading to an impaired awareness of stimuli on the left despite intact early visual processing (Rees et al., 2000). We consider the behavioral manifestations of visual neglect in a later section. Before we do so, we turn from disconnections to a subtler form of computational pathology.

## Uncertainty, Precision, And Autism

### Types of Uncertainty

In predictive coding, the significance ascribed to a given prediction error is determined by the precision of the mapping from hidden causes to the data. If this mapping is very noisy, the gain of the prediction error signal is turned down. A very precise relationship between causes and data leads to an increase in this gain – it is this phenomenon that has been associated with attention (Feldman and Friston, 2010). In other words, attention is the process of affording a greater weight to reliable information.

The generative models depicted in **Figure 1** indicate that there are multiple probability distributions that may be excessively precise or imprecise (Parr and Friston, 2017c). One of these is the sensory precision that relates to the likelihood. It is this that weights sensory prediction errors in predictive coding (Friston and Kiebel, 2009; Feldman and Friston, 2010). Another source of uncertainty relates to the dynamics of hidden causes. It may be that the mapping from the current hidden state to the next is very noisy, or volatile. Alternatively, these transitions may be very deterministic. A third source of uncertainty relates to those states that a person has control over. It is possible for a person to hold beliefs about the course of action, or policy, that they will pursue with differing levels of confidence.

**FIGURE 4:**
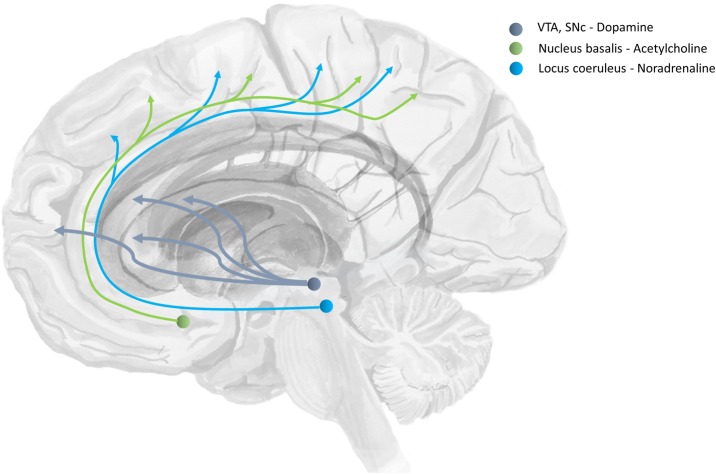
The anatomy of precision. The ascending neuromodulatory systems carrying dopaminergic, cholinergic, and noradrenergic signals are shown (in a simplified form). Dopaminergic neurons have their cell-bodies in the ventral tegmental area (VTA) and the substantia nigra pars compacta (SNc) – two nuclei in the midbrain. The medial forebrain bundle contains the axons of these cells, and allows them to target neurons in the prefrontal cortex and the medium spiny neurons of the striatum. The nucleus basalis of Meynert is found in the basal forebrain. This is the source of cholinergic projections to the cortex (Eckenstein et al., 1988). Axons originating here join the cingulum. Neurons in the locus coeruleus project from the brainstem, through the dorsal noradrenergic bundle, and also join the cingulum to supply the cortex with noradrenaline (Berridge and Waterhouse, 2003).

Beliefs about the degree of uncertainty in each of these three distributions have been related to the transmission of acetylcholine (Dayan and Yu, 2001; Yu and Dayan, 2002; Moran et al., 2013), noradrenaline (Dayan and Yu, 2006), and dopamine (Friston et al., 2014) respectively (Marshall et al., 2016). The ascending neuromodulatory systems associated with these transmitters are depicted in **Figure 4**. The relationship between dopamine and the precision of prior beliefs about policies suggests that the difficulty initiating movements in Parkinson’s disease may be due to a high estimated uncertainty about the course of action to pursue (Friston et al., 2013). A complementary perspective suggests that the role of dopamine is to optimize sequences of actions into the future (O’Reilly and Frank, 2006). Deficient cholinergic signaling has been implicated in the complex visual hallucinations associated with some neurodegenerative conditions (Collerton et al., 2005).

### Precision and Autism

One condition that has received considerable attention using Bayesian formulations is autism (Pellicano and Burr, 2012; Lawson et al., 2014). This condition usefully illustrates how aberrant prior beliefs about uncertainty can produce abnormal percepts. An influential treatment of the inferential deficits in autism argues that the condition can be understood in terms of weak prior beliefs (Pellicano and Burr, 2012). The consequence of this is that autistic individuals rely to a greater extent upon current sensory data to make inferences about hidden causes. This hypothesis is motivated by several empirical observations, including the resistance of people with autism to sensory illusions (Happé, 1996; Simmons et al., 2009), and their superior performance on tasks requiring the location of low-level features in a complex image (Shah and Frith, 1983). The susceptibility of the general population to sensory illusions is thought to be due to the exploitation of artificial scenarios that violate prior beliefs (Geisler and Kersten, 2002; Brown and Friston, 2012). For example, the perception of the concave surface of a mask as a convex face is due to the, normally accurate, prior (or ‘top-down’) belief that faces are convex (Gregory, 1970). Under this prior, the Bayes optimal inference is a false inference (Weiss et al., 2002). If this prior belief is weakened, the optimal inference becomes the true inference.

The excessive dependence on sensory evidence has been described in terms of an aberrant belief about the precision of the likelihood distribution (Lawson et al., 2014). This account additionally considers the source of this belief (Lawson et al., 2017). It suggests that this may be understood in terms of an aberrant prior belief about the volatility of the environment. Volatility here means the ‘noisiness’ (or stochasticity) of the transition probabilities that describe the dynamics of hidden causes in the world. Highly volatile transitions prevent the precise estimation of current states from the past, and result in imprecise beliefs about hidden causes. In other words, past beliefs become less informative when making inferences about the present. Sensory prediction errors then elicit a greater change in beliefs than they would do if a strong prior were in play. This theory of autism has been tested empirically (Lawson et al., 2017), providing a convincing demonstration of computational neuropsychology in practice. Using a Bayesian observer model (Mathys, 2012), it was shown that participants with autism overestimate the volatility of their environment. Complementing this computational finding, pupillary responses, associated with central noradrenergic activity (Koss, 1986), were found to be of a smaller magnitude when participants encountered surprising stimuli compared to neurotypical individuals.

A failure to properly balance the precision of sensory evidence, in relation to prior beliefs, may be a ubiquitous theme in many neuropsychiatric disorders. A potentially important aspect of this imbalance is a failure to attenuate sensory precision during self-made acts. The attenuation of sensory precision is an important aspect of movement and active sensing, because it allows us to temporarily suspend attention to sensory evidence that we are not moving (e.g., in the bradykinesia of Parkinson’s disease). In brief, a failure of sensory attenuation would have profound consequences for self-generated movement, a sense of agency and selfhood. We now consider the implications of Bayesian pathologies for the active interrogation of the sensorium and its neuropsychology.

## Active Inference and Visual Neglect

### Active Sensing

In the above, we have considered how hypotheses are evaluated as if sensory data is passively presented to the brain. In reality, perception is a much more active process of hypothesis testing (Krause, 2008; Yang et al., 2016a,b). Not only are hypotheses formed and refined, but experiments can be performed to confirm or refute them. Saccadic eye movements offer a good example of this, as they turn vision from a passive to an active process (Gibson, 1966; Ognibene and Baldassare, 2015; Parr and Friston, 2017a). Each saccade can be thought of as an experiment to adjudicate between plausible hypotheses about the hidden causes that give rise to visual data (Friston et al., 2012a; Mirza et al., 2016). As in science, the best experiments are those that will bring about the greatest change in beliefs (Lindley, 1956; Friston et al., 2016b; Clark, 2017). A mathematical formulation of this imperative (Friston et al., 2015) suggests that the form of the neuronal message passing required to evaluate different (saccadic) policies maps well to the anatomy of cortico-subcortical loops involving the basal ganglia (Friston et al., 2017d). This is consistent with the known role of this set of subcortical structures in action selection (Gurney et al., 2001; Jahanshahi et al., 2015), and their anatomical projections to oculomotor areas in the midbrain (Hikosaka et al., 2000). To illustrate the importance of these points, we consider visual neglect, a disorder in which active vision is impaired.

### Visual Neglect

A common neuropsychological syndrome, resulting from damage to the right cerebral hemisphere, is visual neglect (Halligan and Marshall, 1998). This is characterized by a failure to attend to the left side of space. This rightward lateralization may be a consequence of the mean-field factorization discussed earlier. Although space is represented bilaterally in the brain, there is no need for representations of identity to be bilateral. This means that the relationships between location and identity should be asymmetrical, complementing the observation that visual neglect is very rarely the consequence of a left hemispheric lesion.

**FIGURE 5:**
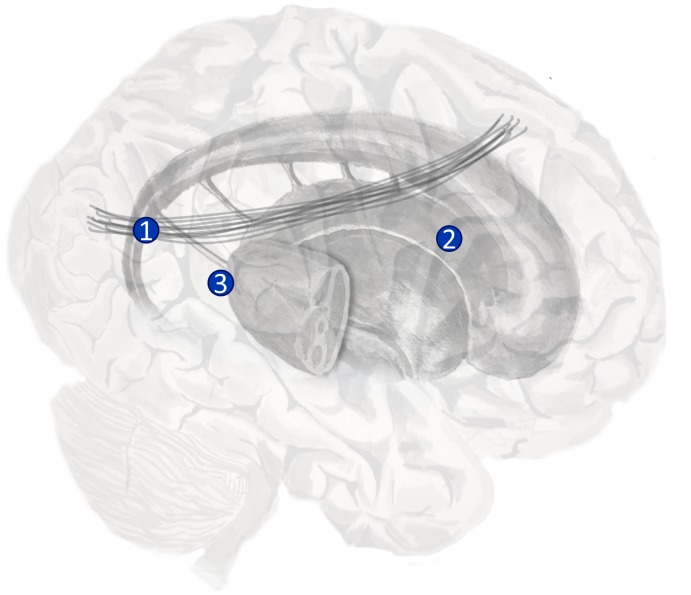
The anatomy of visual neglect. Three lesions implicated in visual neglect are highlighted here. 1 – Disconnection of the second branch of the right superior longitudinal fasciculus (a white matter tract that connects dorsal frontal with ventral parietal regions); 2 – Unilateral lesion to the right putamen; 3 – Unilateral lesion to the right pulvinar (a thalamic nucleus). Note that lesion 1 here is the same as lesion 2 in **Figure 3**.

A behavioral manifestation of this disorder is a bias in saccadic sampling (Husain et al., 2001; Fruhmann Berger et al., 2008; Karnath and Rorden, 2012). Patients with neglect tend to perform saccades to locations on the right more frequently than to those on the left. There are several different sets of prior beliefs that would make this behavior optimal. We will discuss three possibilities (Parr and Friston, 2017b), and consider their biological bases (**Figure 5**). One is a prior belief that proprioceptive data will be consistent with fixations on the right of space. The dorsal parietal lobe is known to contain the ‘parietal eye fields’ (Shipp, 2004), and it is plausible that an input to this region may specify such prior beliefs. A candidate structure is the dorsal pulvinar (Shipp, 2003). This is a thalamic nucleus implicated in attentional processing (Ungerleider and Christensen, 1979; Robinson and Petersen, 1992; Kanai et al., 2015). Crucially, lesions to this structure have been implicated in neglect (Karnath et al., 2002).

A second possibility relates more directly to the question of good experimental design. If a saccade is unlikely to induce a change in current beliefs, then there is little value in performing it. One form that current beliefs take is the likelihood distribution mapping ‘where I am looking’ to ‘what I see’ (Mirza et al., 2016). As illustrated in **Figure 3** (right) this likelihood distribution takes the form of a connection between dorsal frontal and ventral parietal regions (Parr and Friston, 2017a). To adjust beliefs about this mapping, observations could induce a plastic change in synaptic strength following each saccade (Friston et al., 2016b). If the white matter tract connecting these areas is lesioned, it becomes impossible to update these beliefs. As such, if we were to cut the second branch of the superior longitudinal fasciculus (SLF II) on the right, disconnecting dorsal frontal from ventral parietal regions, we would expect there to be no change in beliefs following a saccade to the left. These would make for very poor ‘visual experiments’ (Lindley, 1956). A very similar argument has been put forward for neglect of personal space that emphasizes proprioceptive (rather than visual) consequences of action (Committeri et al., 2007). In these circumstances, optimal behavior would require a greater frequency of rightward saccades. Lesions to SLF II (Doricchi and Tomaiuolo, 2003; Thiebaut de Schotten et al., 2005; Lunven et al., 2015), and the regions it connects (Corbetta et al., 2000; Corbetta and Shulman, 2002, 2011) are associated with neglect.

A third possibility is that the process of policy selection may be inherently biased. Above, we suggested that these computations may involve subcortical structures. The striatum, an input nucleus to the basal ganglia, is well known to be involved in habit formation (Yin and Knowlton, 2006; Graybiel and Grafton, 2015). Habits may be formalized as a bias in prior beliefs about policy selection (FitzGerald et al., 2014). It is plausible that a lesion in the striatum might induce a similar behavioral bias toward saccades to rightward targets. One of the subcortical structures most frequently implicated in visual neglect is the putamen (Karnath et al., 2002), one of the constituent nuclei of the striatum. Such lesions may be interpretable as disrupting the prior belief about policies.

### Anosognosia

The ideas outlined above, that movements can be thought of as sensory experiments, are not limited to eye movements and visual data. Plausibly, limb movements could be used to test hypotheses about proprioceptive (and visual) sensations. This has interesting consequences for a neuropsychological deficit known as anosognosia (Fotopoulou, 2012). This syndrome can accompany hemiplegia, which prevents the performance of perceptual experiments using the paralyzed limb (Fotopoulou, 2014). In addition to the failure to perform such an experiment, patients must be able to ignore any discrepancy between predicted movements and the contradictory sensory data suggesting the absence of a movement (Frith et al., 2000). As this failure of monitoring movement trajectories can be induced in healthy subjects (Fourneret and Jeannerod, 1998), it seems plausible that this could be exaggerated in the context of hemiplegia, through a dampening of exteroceptive sensory precision.

This explanation is not sufficient on its own, as anosognosia does not occur in all cases of hemiplegia. Lesion mapping has implicated the insula in the deficits observed in these patients (Karnath et al., 2005; Fotopoulou et al., 2010). This is a region often associated with interoceptive inference (Barrett and Simmons, 2015) that has substantial efferent connectivity to somatosensory cortex (Showers and Lauer, 1961; Mesulam and Mufson, 1982). Damage to the insula and surrounding regions might reflect a disconnection of the mapping from motor hypotheses to the interoceptive data that accounts for what it ‘feels like’ to move a limb. This is consistent with evidence that the insula mediates inferences about these kinds of sensations (Allen et al., 2016). A plausible hypothesis for the computational pathology in anosognosia is then that a failure of *active* inference is combined with a disconnection of the likelihood mapping between motor control and its interoceptive (and exteroceptive) consequences (Fotopoulou et al., 2008).

## A (Provisional) Taxonomy of Computational Pathology

In the above, we have described the components of a generative model required to perform Bayesian inference. We have reviewed some of the syndromes that may illustrate deficits of one or more of these components. Broadly, the generative model constitutes beliefs about the hidden states, their dynamics, and the mechanisms by which sensory data is generated from hidden states. Each of these beliefs can be disrupted through an increase or decrease in precision, or through disconnections. Modulation of precision implicates the ascending neuromodulatory systems. This modulation may be important for a range of neuropsychiatric and functional neurological disorders (Edwards et al., 2012).

In addition to modulation of connectivity, disconnections can completely disrupt beliefs about the conditional probability of one variable given another. The hierarchical architecture of the cortex suggests that inter-areal white matter tracts, the most vulnerable to vascular or inflammatory lesions, represent likelihood distributions (i.e., the probability of data, or a low-level cause, given a high-level cause). Drawing upon the notion of a mean-field factorization, we noted that such disconnections are likely to have a hemispheric asymmetry in the behaviors they elicit. It is also plausible that functional disconnections might occur within a cortical region. This would allow for disruption of transition probabilities. While not as vulnerable to vascular insult, other pathologies can cause changes in intrinsic cortical connectivity (Cooray et al., 2015).

Epistemic, foraging, behavior is vital for the evaluation of beliefs about the world. Unusual patterns of sensorimotor sampling can be induced by abnormal beliefs about the motor experiments that best disambiguate between perceptual hypotheses. These computations implicate subcortical structures, such as the basal ganglia. There are two ways that disruption of these computations may result in abnormal behavior. The first is that prior beliefs about policies may be biased. This can be an indirect effect, through other beliefs, or a direct effect due to dysfunction in basal ganglia networks. The second is that an impairment in performing these experiments, due to paralysis, might impair the refutation of incorrect perceptual hypotheses. This may be compounded by a disconnection or a neuromodulatory failure, as has been proposed in anosognosia.

One further source of an aberrant priors, not discussed in the above, is neuronal loss. In neurodegenerative disorders, there may be a reduction in the number of neurons in a given brain area. This results in a smaller number of possible activity patterns across these neurons and limits the number of hypotheses they can represent. This means that disorders in which neurons are lost may cause a shrinkage of the brain’s hypothesis space. In other words, the failure to form accurate perceptual hypotheses in such conditions may be due to an attrition of the number of hypotheses that can be entertained by the brain. An important future step in Bayesian neuropsychology will be linking tissue pathology with computation more directly. This may be one route toward achieving this.

## Conclusion

While Bayesian approaches are not in conflict with other methods in computational neuroscience, they do offer a different (complementary) perspective that is often very useful. For example, many traditional modeling approaches would not predict that disconnections in early sensory streams, such as the retino-geniculate system, could result in complex sensory hallucinations. Calling upon a hierarchical generative model that makes ‘top-down’ predictions about sensory data, clarifies and provides insight into such issues. In the above we have discussed the features of the generative models that underwrite perception and behavior. We have illustrated the importance of these features through examples of their failures. These computational pathologies can be described in terms of abnormal prior beliefs, or in terms of their biological substrates. We noted that aberrant priors about the structure of a likelihood mapping relate to disconnection syndromes, ubiquitous in neurology. Pathological beliefs about uncertainty may manifest as neuromodulatory disorders. The process of identifying the pathological priors that give rise to Bayes optimal behavior in patients is promising both scientifically and clinically. If individual patients can be uniquely characterized by subject-specific priors, this facilitates a precision medicine approach grounded in computational phenotyping (Adams et al., 2016; Schwartenbeck and Friston, 2016; Mirza et al., 2018). This also allows for empirical evaluation of hypotheses about abnormal priors, by comparing quantitative, computational phenotypes between clinical and healthy populations. Relating these priors to their biological substrates offers the further possibility of treatments that target aberrant neurobiology in a patient specific manner.

## Author Contributions

All authors listed have made a substantial, direct and intellectual contribution to the work, and approved it for publication.

## Conflict of Interest Statement

The authors declare that the research was conducted in the absence of any commercial or financial relationships that could be construed as a potential conflict of interest.
